# FDG‐PET patterns associate with survival in patients with prion disease

**DOI:** 10.1002/acn3.52230

**Published:** 2024-10-29

**Authors:** Nick Corriveau‐Lecavalier, Yoav D. Piura, Brian S. Appleby, Dror Shir, Leland R. Barnard, Venkatsampath Gogineni, David T. Jones, Gregory S. Day

**Affiliations:** ^1^ Department of Neurology Mayo Clinic Rochester Minnesota USA; ^2^ Department of Psychiatry and Psychology Mayo Clinic Rochester Minnesota USA; ^3^ Department of Neurology Mayo Clinic in Florida Jacksonville Florida USA; ^4^ National Prion Disease Pathology Surveillance Center, Case Western Reserve Cleveland Ohio USA; ^5^ Department of Radiology Mayo Clinic Rochester Minnesota USA

## Abstract

**Objective:**

Prion disease classically presents with rapidly progressive dementia, leading to death within months of diagnosis. Advances in diagnostic testing have improved recognition of patients with atypical presentations and protracted disease courses, raising key questions surrounding the relationship between patterns of neurodegeneration and survival. We assessed the contribution of fluorodeoxyglucose (FDG‐PET) imaging for this purpose.

**Methods:**

FDG‐PET were performed in 40 clinic patients with prion disease. FDG‐PET images were projected onto latent factors generated in an external dataset to yield patient‐specific eigenvalues. Eigenvalues were input into a clustering algorithm to generate data‐driven clusters, which were compared by survival time.

**Results:**

Median age at FDG‐PET was 65.3 years (range 23–85). Median time from FDG‐PET to death was 3.7 months (range 0.3–19.0). Four data‐driven clusters were generated, termed “Neocortical” (*n* = 7), “Transitional” (*n* = 12), “Temporo‐parietal” (*n* = 13), and “Deep nuclei” (*n* = 6). Deep nuclei and transitional clusters had a shorter survival time than the neocortical cluster. Subsequent analyses suggested that this difference was driven by greater hypometabolism of deep nuclei relative to neocortical areas. FDG‐PET‐patterns were not associated with demographic (age and sex) or clinical (CSF total‐tau, 14‐3‐3) variables.

**Interpretation:**

Greater hypometabolism within deep nuclei relative to neocortical areas associated with more rapid decline in patients with prion disease and vice versa. FDG‐PET informs large‐scale network physiology and may inform the relationship between spreading pathology and survival in patients with prion disease. Future studies should consider whether FDG‐PET may enrich multimodal prion disease prognostication models.

## Introduction

Prion disease is a rare and uniformly fatal prion disease that classically presents with rapidly progressive dementia, culminating in death within months of the diagnosis.^1^, ^2^, ^3^ Expanded access to real‐time quaking‐induced conversion (RT‐QuIC) assays capable of detecting small amounts of prions in patient specimens has enabled antemortem diagnoses with high sensitivity and specificity—particularly when interpreted together with other established markers of prion disease, including CSF total tau (T‐Tau) and brain MRI.^4^, ^5^, ^6^, ^7^, ^8^, ^9^, ^10^, ^11^, ^12^, ^13^, ^14^, ^15^ Broad application of these measures has facilitated broader recognition of patients,^8^, ^9^, ^10^, ^16^ supporting diagnoses at earlier (even preclinical) disease stages.^17^, ^18^ Importantly, this earlier detection has enabled the recognition of patients with atypical clinical phenotypes,^14^ including those with clinical courses ranging from weeks to years.^1^, ^3^, ^19^, ^20^, ^21^ Greater appreciation of the full spectrum of prion cases raises questions concerning the patient‐ and disease‐specific factors that contribute to variability in the clinical presentation and rates of decline. The answers to these questions may inform clinical counseling and potentially improve selection/allocation of patients within clinical trials of putative disease‐modifying therapies for prion disorders.^22^

Several clinical and biological parameters have been assessed in relation to disease duration in prion disease (specifically in Creutzfeldt–Jacob disease or CJD), including age at symptom onset, sex, codon 129 genotype, PrP^Sc^ molecular subtype,^19^, ^23^, ^24^ clinical presentation,^4^, ^25^ and CSF‐ and blood‐based biomarkers of neurodegeneration (i.e., neurofilament chain and total‐Tau, 14‐3‐3).^4^, ^24^, ^26^, ^28^ Multimodal models specifically dedicated to survival time prediction have been developed in recent years.^24^, ^29^ These models achieved areas under the curve ranging from 0.67 to 0.94 using a combination of demographic (age and sex), codon 129 genotype, clinical phenotype, MRI abnormalities, and scores on various clinical scales.^24^, ^29^ Performance characteristics emphasize the ability of these models to capture disease‐ and patient‐specific variables that influence the rate of clinical progression. Although useful, these models do not depict the localization of prion pathology, neuronal dysfunction, or neurodegeneration at the level of the individual patient that is presumed to contribute to heterogeneity in the clinical presentation and course of prion disease.^30^, ^31^, ^32^, ^33^

Patterns of network degeneration may serve as intermediate phenotypes between molecular pathophysiology and clinical manifestations across common neurodegenerative diseases.^34^, ^35^ This principle may extend to prion disease, noting significant associations between disease duration and the extent of basal ganglia abnormality on DWI and T2‐FLAIR MR imaging^36^, ^37^, ^38^ and between regional MRI volumetric data and MMSE scores^39^ in patients with sporadic CJD. However, MRI tends to reflect accumulated disease‐related changes, being relatively insensitive to dynamic disease processes.^40^, ^41^ Fluorodeoxyglucose (FDG)‐PET imaging is a more direct measure of active, dynamic disease processes seen with neurodegenerative disease,^34^, ^42^ with the ability to track disease progression in most common neurodegenerative disease such as Alzheimer disease (AD)^43^, ^44^ and frontotemporal lobar degeneration.^45^ FDG‐PET can also objectively index phenotypic heterogeneity,^30^, ^31^, ^32^, ^33^ with patterns of hypometabolism reflecting post‐mortem prion accumulation in patients with sporadic CJD^46^, ^47^ and time to symptom onset in patients with fatal familial insomnia.^48^ As such, FDG‐PET may inform unique aspects of survival by revealing associations between distinct topologies of prion disease and neurodegeneration. This could provide additional insight into disease pathogenesis that cannot be reliably inferred from biofluid biomarkers of neurodegeneration, including total tau, 14‐3‐3, and neurofilament light chain.^24^, ^26^, ^27^, ^49^, ^50^ With this in mind, we assessed whether patterns of network degeneration assessed with FDG‐PET inform survival. Specifically, we applied advanced data analytics to generate data‐driven metabolic clusters from FDG‐PET images and assessed the association between clusters, disease duration (i.e., time from FDG‐PET to death), and other relevant clinical variables in patients diagnosed with prion disease at our tertiary care center. We also considered how patterns of relative hyper‐ versus hypometabolism contributed to these relationships. Finally, we performed an exploratory analysis to assess how quantitative FDG‐PET analysis compared to established changes on MRI in predicting survival time.

## Methods

### Participants

Clinical and imaging data were collected from 40 patients with prion disease who were evaluated and diagnosed from December 2006 to May 2023 as part of routine clinical care across the Mayo Clinic Enterprise (Rochester *n* = 36, Florida *n* = 3, and Arizona *n* = 1). Diagnoses of probable CJD were attributed by expert neurologists, integrating findings from clinical history, neurological examination, structural neuroimaging (MRI), electroencephalography, and CSF evaluation, as previously described.^4^, ^5^, ^51^, ^52^ Definite prion disease was diagnosed in patients with neuropathological confirmation of protease‐resistant PrP. An aliquot of CSF was sent to the National Prion Disease Pathology Surveillance Center (NPDPSC) (Case Western Reserve University; Cleveland, OH) for RT‐QuIC testing for abnormal prion seeding activity and T‐tau and 14‐3‐3 measures.^9^ CSF T‐tau levels greater than 1149 pg/mL were considered consistent with CJD.^9^, ^31^, ^53^ A predominant clinical phenotype was retrospectively attributed for each patient with prion disease patient by expert behavioral neurologists (D.T.J., D.S., and G.S.D.) and included cognitive, motor, psychiatric, global, and cerebellar presentations.^4^ Death date and results of neuropathological evaluation and molecular subtyping as provided by the NPDPSC were recorded when available. When possible, autopsy cases were classified according to their histo‐molecular subtype, according to current classification criteria.^54^ Antemortem measures of *PRNP* genotype were not routinely performed in clinic patients, nor were patients enrolled in research protocols permitting banking of DNA. Consequently, codon 129 variant status was unknown in most patients who did not undergo autopsy.

To be included in the study, patients had to have available FDG‐PET imaging. FDG‐PET imaging was ordered by experienced behavioral neurologists to clarify the differential diagnosis in patients with unexplained cognitive impairment and was generally completed following the initial clinic visit, before disease‐specific biomarker results (i.e., RT‐QuIC) were returned. Imaging findings in patients with prion disease (*n* = 40) were compared against those from 39 age‐ and sex‐matched cognitively normal (“control”) participants assessed and imaged via the Mayo Clinic Study of Aging—an epidemiologic study of older community‐dwelling adults within Olmstead County, Minnesota.^55^ Control participants were amyloid‐ and tau‐negative on PET imaging.

### Standard protocols approvals, registrations, and patient consents

This study was approved by the Mayo Clinic Institutional Review Board. A waiver of consent was granted for the use of deidentified data obtained during clinical evaluation. This study followed the Strengthening the Reporting of Observational Studies in Epidemiology (STROBE) reporting guideline for cohort studies.

### FDG‐PET imaging

FDG‐PET images were acquired using a PET/CT scanner (GE Healthcare) following a 30‐min uptake period while waiting in a dimly lit room. The scanning session lasted 8 min divided into four 2‐min dynamic frames following a low‐dose CT transmission scan. Images were processed using an MRI‐free pipeline involving the registration of the FDG‐PET images to the Mayo Clinic Adult Lifespan Template space (https://www.nitrc.org/projects/mcalt/) using a nonlinear symmetric diffeomorphic registration. FDG‐PET were intensity normalized to the pons and smoothed using a 6‐mm full‐width at half‐maximum kernel.

We performed a spectral decomposition of FDG‐PET images by projecting them onto a latent space generated in an independent dataset and externally applied in a wide range of neurodegenerative dementing illnesses using previously described methods.^34^, ^56^ Briefly, this technique consists in assessing the covariance similarity between FDG‐PET images from each patient with prion disease to 150 latent factors (referred to as “eigenbrains”) derived from a principal component analysis performed in large dataset of patients spanning the normal aging and AD spectrum (*n* = 998). These eigenbrains represent a relative distribution of metabolism across the entire brain with opposing poles of relative hypo‐ and hyper‐metabolism. This procedure yields 150 “eigenvalues” per patient that represent the extent to which a patient‐specific FDG‐PET image resembles the metabolism pattern expressed by each eigenbrain. These individual eigenvalues can then be used for subsequent analyses.

### MRI imaging

MRI studies were performed in clinical settings using various protocols on 1.5 T or 3 T machines. We calculated an MRI ratio score measuring the relative neocortical‐to‐deep nuclei involvement across patients with prion disease. Two authors (GSD and YDP) reviewed diffusion weighted and acute diffusion coefficient images to define areas of diffusion restriction affecting neocortical (parietal, occipital, temporal, posterior cingulate, and insula) and deep nuclei regions (caudate, putamen, globus pallidum, and thalamus). A value of 1 was assigned to regions with diffusion restriction; a value of 0 was assigned to unaffected regions. Reviews were conducted independently, blinded to FDG‐PET data. We then calculated the average score for neocortical areas and for deep nuclei areas for each patient. The two average scores were summed to generate a neocortical‐to‐deep nuclei ratio, with neocortical composite having a positive weight and deep nuclei composite having a negative weight. Here, a negative score means greater deep nuclei involvement relative to neocortical areas, and vice versa.

### Statistical analyses

Analyses were conducted using *R* version 4.3.2 and Python 3.7. We first performed a hierarchical spectral clustering analysis on eigenvalues of the 150 eigenbrains across the prion disease cohort using the *apcluster* package. We created patient‐by‐patient similarity matrices based on several distance metrics (Euclidean, Manhattan, Canberra, and Minkowski) applied to scaled eigenvalues. Each matrix was submitted to an affinity propagation clustering algorithm, which is based on the identification of “exemplar” patients and the formation of clusters around these exemplars, which is computed based on the optimization of within‐ versus between‐cluster similarity.^57^ The optimal clustering solution was determined by comparing silhouette coefficient values across distance metrics.^58^

We performed pair‐wise comparisons between voxel‐wise patterns of FDG‐PET metabolism of each prion disease cluster derived from the hierarchical clustering analysis to those of age‐ and sex‐matched cognitively normal (“control”) participants. This consisted in calculating the mean and standard deviation of each voxel of the brain for each group (prion disease clusters and controls), and then calculating the *Z* score of each voxel in a given prion disease cluster relative to the mean image of the control group, resulting in whole‐brain voxel‐wise *Z* score maps. We then compared demographic (age at FDG‐PET and symptom onset, and sex), clinical (months from symptom onset or FDG to death), and biomarker data (CSF T‐Tau and 14‐3‐3 levels) between prion disease clusters using ANOVAs with Tukey's test for post‐hoc comparisons for continuous variables and chi‐squared analyses for categorical variables.

We then conducted a follow‐up analysis to assess which patterns of whole‐brain metabolism (eigenbrains) used as input features in the hierarchical clustering algorithm most contributed to the formation of prion disease clusters. This was done by comparing prion disease clusters on eigenvalues using ANOVAs with Tukey's post hoc test for post hoc comparisons. This was restricted to the first 10 eigenbrains. We then examined the relationship between eigenvalues of eigenbrains showing significant differences and months from FDG‐PET to death using linear regressions. We then performed a linear regression model to examine the relationship between months from MRI to death and the MRI ratio score. This model was then compared with FDG models (eigenvalues and months from FDG‐PET to death) using ANOVAs.

## Results

### Demographic and clinical data

Demographic and clinical details are summarized in Table 1. Age at symptom onset was broad (median 64.4 years; range 21.9–81.4), with a similar distribution of males and females. Thirty‐nine patients were non‐Hispanic White; one patient was Hispanic White. The cohort adequately reflected the breadth of clinical presentations of prion disease, including patients with amnestic (*n* = 4), aphasic (*n* = 1), corticobasal (*n* = 2), cerebellar (*n* = 7), dysexecutive (*n* = 5), extrapyramidal (*n* = 1), global (*n* = 9), hyperkinetic (*n* = 2), psychiatric (*n* = 2), and visual/Heidenhain (*n* = 7) phenotypes. FDG‐PET was performed at a median of 6.4 months from symptom onset (range 0.2–41.5 months) to clarify the cause of cognitive impairment. At the time of analyses, nearly all patients (39/40) had succumbed to their illness, with median symptomatic duration 13.0 months (range 1.0–53.3). The median number of months from FDG to death was 3.7 (range 0.3–19.2). One patient had a low quality FDG‐PET scan and was excluded from subsequent analyses. Molecular subtype was available from 18/40 patients with definite prion disease confirmed via autopsy examination or genetic testing. Subtypes of sporadic CJD were distributed as follows: MM1 (*n* = 4), MM2 (*n* = 4), MV2C (*n* = 2), VV2 (*n* = 2), MM1‐2 (*n* = 1), MV1‐2C (*n* = 1), and VV1‐2 (*n* = 1). Two patients had genetic CJD associated with E200K‐129 M mutations. One patient with a predominant psychiatric presentation and low CSF T‐tau levels (78 pg/mL) had sporadic fatal insomnia, confirmed at autopsy (MM2T). Supplementary Table 1 includes patient‐level characterization of autopsy cases.

**Table 1:** Demographic and clinical details in patients with prion disease.

Measure	*n* = 40
Patient demographics	
Age at symptom onset, median years (range)	64.4 (21.9–81.4)
Age at FDG‐PET, median years (range)	65.3 (22.8–84.9)
Female sex (%)	21 (53)
Presenting symptoms and signs	
Rapidly progressive dementia (%)	38 (95)
Predominant clinical phenotype	
Amnestic (%)	5 (13)
Aphasia (%)	1 (3)
Corticobasal (%)	2 (5)
Cerebellar (%)	7 (18)
Dysexecutive (%)	4 (10)
Extrapyramidal (%)	1 (3)
Global (%)	10 (25)
Hyperkinetic (%)	2 (5)
Psychiatric (%)	2 (5)
Visual (%)	7 (18)
Diagnostic testing	
MRI findings compatible with CJD^a^ (%)	38 (95)
Location of MRI findings	
Caudate/putamen (%)	21 (53)
Thalamus (%)	11 (28)
Temporal (%)	17 (43)
Parietal (%)	24 (63)
Occipital (%)	15 (38)
Months from symptom onset to MRI, median (range)	6.1 (0.2–41)
Cerebrospinal fluid testing compatible with CJD (%)	40 (100)
RT‐QuIC, positive (%)	33/36 (92)
14‐3‐3, positive	30/39 (77)
Total Tau >1149 pg/mL (%)	33/38 (87)
Months from symptom onset to CSF, median (range)	6.6 (0.3–41.8)
Total Tau pg/mL, median (range)	3715 (2007–5386)

Continuous variables are expressed as median and range.

CJD, Creutzfeldt–Jakob disease; FDG, fluorodeoxyglucose; RT‐QuIC, real‐time quaking‐induced conversion.

^a^ Brain MRI criteria as outlined within the Diagnostic Criteria by the Centers for Disease Control and Prevention (high signal in caudate or putamen or two cortical regions [temporal, parietal, and occipital] either on diffusion‐weighted imaging or fluid‐attenuated inversion recovery).^20^

### Data‐driven metabolic clusters

The optimal clustering solution was based on the Euclidean distance and generated five distinct data‐driven clusters. One cluster included only one patient with predominant extrapyramidal symptoms and a pattern of mixed frontal hypometabolism and temporo‐occipital hypermetabolism. Specific data for this patient are presented in Supplementary Figure 1. Main analyses were focused on the four remaining clusters.

**Figure 1:**
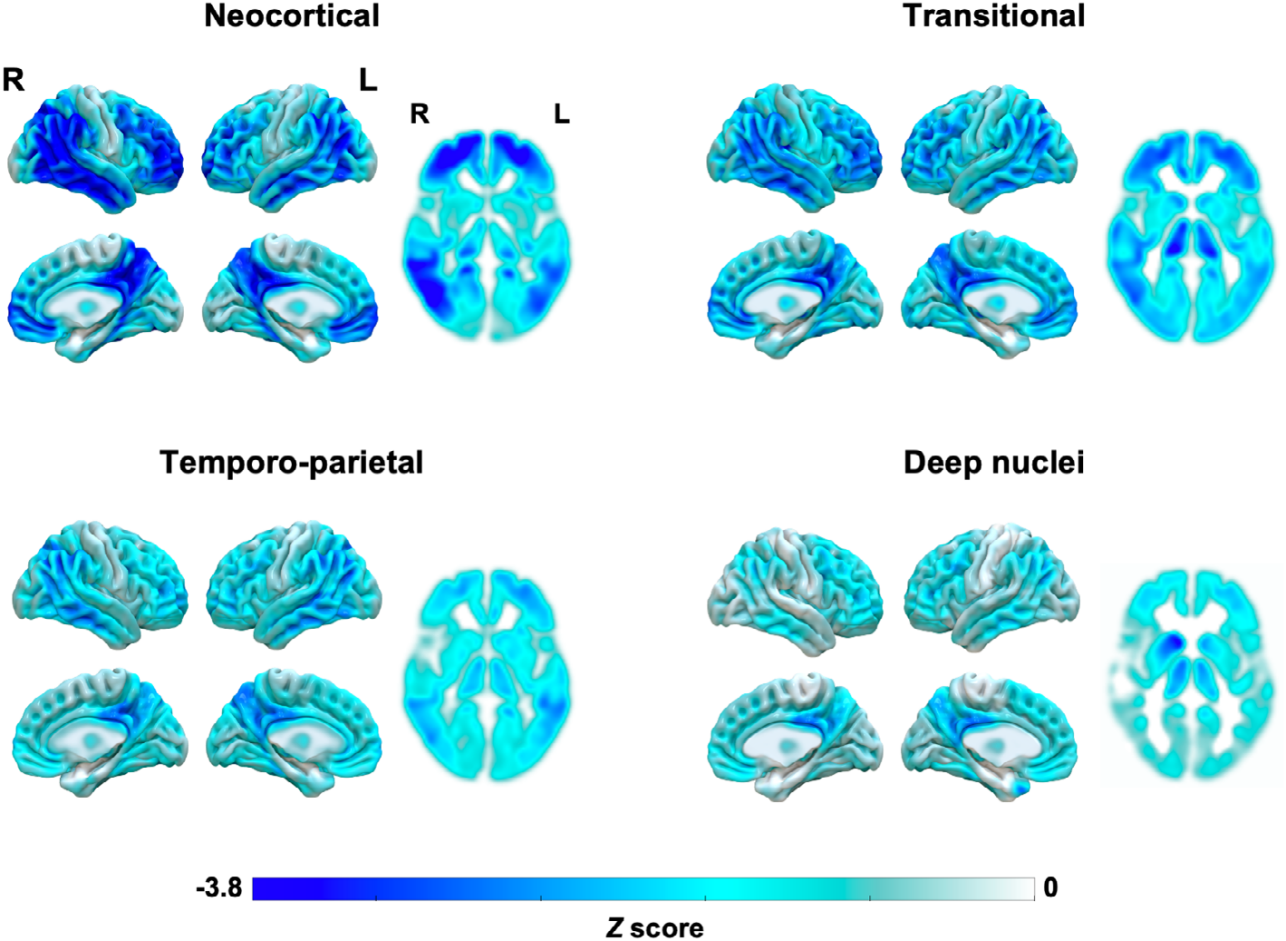
Data‐driven metabolic clusters. Patterns of FDG‐PET metabolism for each cluster were compared to a group of age‐ and sex‐matched cognitively unimpaired individuals. Values are expressed as *Z‐*scores at the voxel‐level. Whole‐brain renderings were generated using SurfIce (https://www.nitrc.org/projects/surfice/) and the 2D slices at the deep nuclei level were generated using MRIcroGL (https://www.nitrc.org/projects/mricrogl). FDG = fluorodeoxyglucose.

The four clusters were termed “Neocortical” (*n* = 7), “Transitional” (*n* = 12), “Temporo‐parietal” (*n* = 13), and “Deep nuclei” (*n* = 6) according to their pattern of whole‐brain hypometabolism relative to cognitively normal controls (Fig. 1). The neocortical cluster had prominent hypometabolism spanning frontal, parietal, and lateral occipito‐temporal areas with minimal deep nuclei hypometabolism. The transitional cluster had a mixed pattern with neocortical (prefrontal, parietal, and lateral temporal) and thalamic hypometabolism. The temporo‐parietal cluster showed predominant hypometabolism in parietal and lateral temporal areas with minimal deep nuclei hypometabolism. In contrast, the deep nuclei cluster had prominent thalamic, caudate, putamen, and globus pallidum involvement with a slight right hemispheric predominance with little neocortical hypometabolism aside from mild bilateral precuneus hypometabolism.

**Figure 2:**
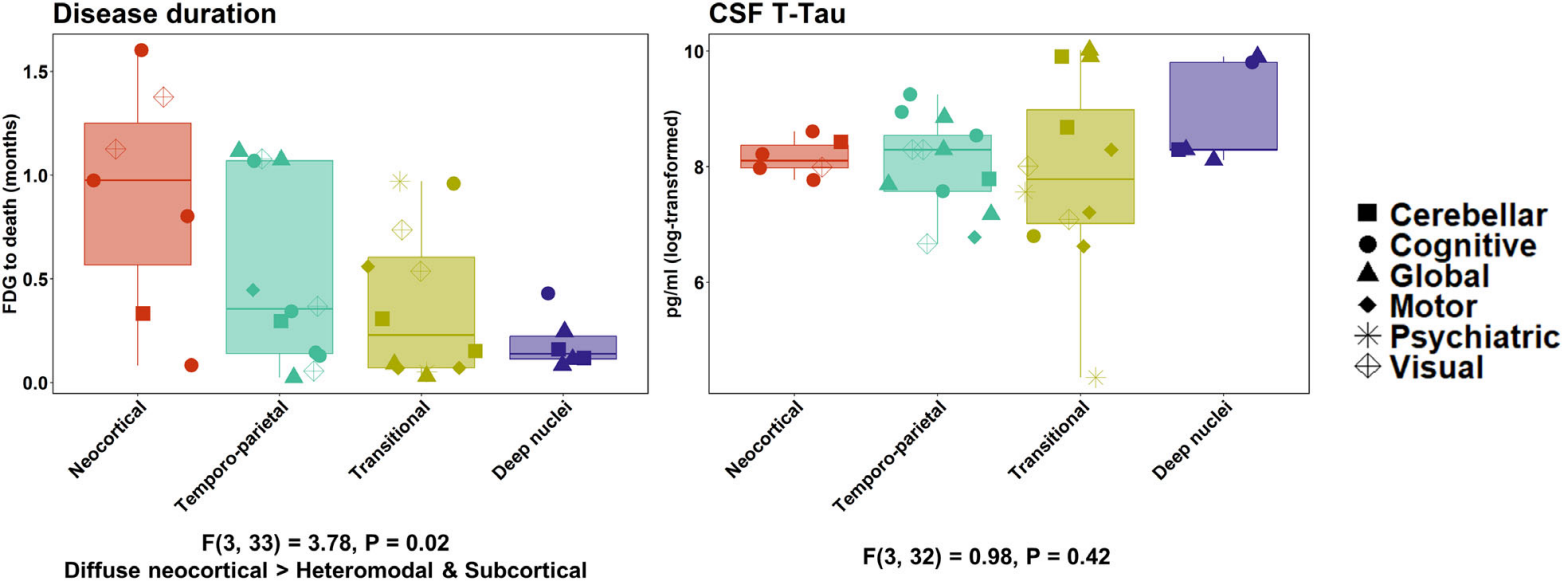
Cluster comparisons on markers of disease progression. Dot shapes indicate predominant phenotypes grouped as cognitive (amnesic, aphasia, and dysexecutive), motor (corticobasal, extrapyramidal, and hyperkinetic), global, cerebellar, psychiatric, and visual. FDG = fluorodeoxyglucose; T‐Tau = total‐tau.

Comparisons of prion disease clusters on demographic and clinical variables are summarized in Table 2. Clusters differed in terms of months between FDG‐PET and death. Patients within the deep nuclei (median 1.7, range 1.0–5.2) and transitional (median 2.8, range 0.4–11.6) clusters had shorter disease duration relative to patients within the neocortical cluster (median 11.7, range 1.0–19.2). Clusters did not differ on age at symptom onset, months from symptom onset to FDG‐PET, sex distribution, or CSF biomarkers (i.e., 14‐3‐3, T‐Tau). Figure 2 depicts differences between clusters in months from FDG‐PET to death (i.e., survival) and CSF biomarkers.

**Table 2:** Data‐driven metabolic clusters.

	Neocortical	Transitional	Temporo‐parietal	Deep nuclei	*p*
*n*	7	12	13	6	–
Age at symptom onset, median years (range)	63.6 (51.6–81.4)	63.1 (1.9–76.9)	64.7 (44.1–72.0)	65.6 (60.2–74)	0.46
Age at FDG‐PET, median years (range)	64.2 (52.2–84.9)	63.3 (22.8–77.4)	66.4 (44.4–72.3)	63.6 (62.4–74.6)	0.44
Sex at birth (female, male)	5, 2	8, 4	4, 9	4, 2	0.20
Months from symptom onset to death, median (range)	26.8 (4.2–53.3)	13.3 2.28–34	14.8 (0.96–35)	6.2, 2.16–39.7	0.14
Months from symptom onset to FDG, median (range)	8.81 (7.3–18.4)	7.2 (2.1–12.9)	4.1 (2.7–10.7)	4.5 (3.4–6.4)	0.59
Months from symptom onset to lumbar puncture, median (range)	9.1 (7.2–17.7)	6.1 (2.8–13.8)	6.2 (2.6–13.1)	4.5 (2.7–6.0)	0.69
Months from FDG to death, median (range)	11.7 (1.0–19.2)	2.75 (0.4–11.6)	4.26 (0.3–13.4)	1.67 (1.0–5.2)	0.02^a^
Predominant phenotype					
Amnestic	2	0	1	1	–
Aphasic	0	0	1	0	–
Corticobasal	0	2	0	0	–
Cerebellar	1	2	1	2	–
Dysexecutive	2	1	2	0	–
Extrapyramidal	0	0	1	0	–
Global	0	2	4	3	–
Hyperkinetic	0	1	0	0	–
Psychiatric	0	2	0	0	–
Visual	2	2	3	0	–
Cerebrospinal fluid tests for CJD (%)					
RT‐QuIC, positive (%)	6/6 (100)	9/11 (82)	9/10 (90)	5/5 (100)	0.50
14‐3‐3, positive	6/6 (100)	10/12 (83)	8/13 (62)	5/6 (83)	0.50
Total‐Tau >1149 pg/mL (%)	6/6 (100)	9/12 (75)	11/13 (85)	5/6 (83)	0.40
Total‐Tau (pg/mL), median (range)	3329 (2373–5473)	2466 (78–22379)	4000 (785–10413)	4000 (3334–20000)	0.42

Continuous variables are expressed as median and range.

FDG, fluorodeoxyglucose.

^a^ Neocortical > deep nuclei (*p* = 0.018) and transitional (*p* = 0.04).

### Analysis of factors contributing to clustering and survival

**Figure 3:**
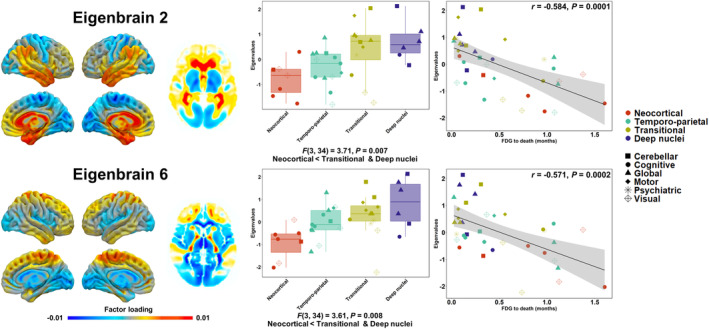
Comparisons of data‐driven metabolic clusters on eigenbrains. The eigenbrains were derived using an independent dataset of research patients as described in Jones et al.^34^ The color bars represent positive (warm colors) and negative (cold colors) loadings associated with each eigenbrain. The eigenbrains reflect relative metabolism between two sets of brain areas, and the directionality (positive and negative) is arbitrary. For both eigenbrains, higher eigenvalues are indicative of lower metabolism in cold colors relative to warm colors, and vice versa. Only eigenbrains showing significant differences across clusters are shown. Dot shapes indicate predominant phenotypes grouped as cognitive (amnesic, aphasia, and dysexecutive), motor (corticobasal, extrapyramidal, and hyperkinetic), global, cerebellar, psychiatric, and visual.

A follow‐up analysis comparing prion disease clusters on the first 10 eigenbrains used as input features for the hierarchical clustering revealed significant differences on eigenbrains 2 and 6 (see Fig. 3). Eigenbrain 2 reflects a distribution of metabolism opposing lateral and medial temporal and orbitofrontal areas to deep nuclei, occipital, and bilateral sensorimotor areas. Here, the deep nuclei and transitional clusters had higher eigenvalues compared to the neocortical cluster. This means that patients within these two clusters had more hypometabolism in deep nuclei, occipital, and sensorimotor areas and preserved temporal and orbitofrontal areas, whereas the neocortical showed the opposite pattern. Eigenbrain 6 reflects a distribution of metabolism opposing lateral temporal, prefrontal, and sensorimotor areas to deep nuclei, insular, and occipital areas. Here, the deep nuclei and transitional clusters also had higher eigenvalues relative to the neocortical cluster. This means that these clusters had more hypometabolism in deep nuclei, insular, and occipital areas and preserved metabolism in temporal, prefrontal and sensorimotor areas, whereas the neocortical showed the opposite pattern.

Assessment of the relationship between eigenvalues for eigenbrains 2 and 6 and months between FDG‐PET and death across the whole prion disease cohort revealed inverse linear associations (*r* = −0.584 *p* = 0.0001 for eigenbrain 2 and *r* = −0.571 *p* = 0.0002 for eigenbrain 6). This means that higher eigenvalues on both eigenbrains (i.e., more hypometabolism in deep nuclei, occipital, and sensorimotor areas relative to temporal and orbitofrontal areas for eigenbrain 2; more hypometabolism in deep nuclei, insular, and visual areas relative to temporal, prefrontal, and sensorimotor areas for eigenbrain 6) were associated with shorter disease duration, and vice‐versa. In comparison, CSF T‐Tau levels did not associate with survival (i.e., time from lumbar puncture to death: *r* = −0.229, *p* = 0.19). Visual depictions of associations between FDG‐PET patterns, data‐driven subtypes, and molecular subtypes are reported in Supplementary Figure 2.

### Exploratory analyses of MRI ratio score and survival

Assessment of the relationship between the MRI ratio score (indicating relative involvement of neocortical versus deep nuclei regions) and months between MRI and death across the prion disease cohort revealed a positive linear association (*r* = 0.474 *p* = 0.003). Greater diffusion restriction within deep nuclei relative to neocortical areas was associated with shorter disease duration, and vice‐versa. The association between MRI ratio score and survival was less than that observed with FDG‐PET models, (*F* (1, 35) = 18.2, *p* < 0.001 compared to eigenbrain 2, *F* (1, 35) = 16.9, *p* < 0.001 compared to eigenbrain 6).

## Discussion

Whereas the ultimate outcome is clear in patients with prion disease, it remains challenging to reliably estimate clinical trajectories in individual patients—particularly early in the symptomatic course. Reliable estimates are essential to inform patient and caregiver plans and to ensure access to appropriate healthcare resources, including timely enrollment in hospice and other end‐of‐life care programs. Existing algorithms have accomplished this by combining clinical and genetic measures with non‐specific markers of neurodegeneration.^24^, ^29^ FDG‐PET findings may also inform clinical trajectories by capturing patterns of network degeneration that associate with survival. In particular, our findings emphasize the privileged role assigned to networks involving deep nuclei. Clusters with worse hypometabolism in deep nuclei, namely the transitional and deep nuclei clusters, had a shorter disease duration relative to the neocortical cluster. Importantly, these differences did not correlate with demographic details (e.g., age and sex) and CSF biomarkers (i.e., T‐tau levels) known to associate with disease duration,^4^, ^19^, ^23^, ^24^, ^26^, ^27^ implying that the localization of neuronal damage may provide unique information on survival.

These findings build upon prior studies reporting accelerated declines in patients with CJD and abnormalities in the basal ganglia on structural MR neuroimaging,^36^, ^37^, ^38^ informing the broader patterns of network degeneration that influence survival. Indeed, our follow‐up analysis of eigenbrains revealed that the relationship between network degeneration and disease duration was not restricted to deep nuclei in isolation, but rather involved whole‐brain patterns of relative hyper‐ and hypometabolism. Thus, restricting analyses to deep nuclei may be suboptimal to estimate whether a patient will experience a rapid or more protracted course—PET interpretations must account for whole‐brain patterns of relative hypo‐ versus hypermetabolism. In addition, our exploratory analysis suggested that these data‐driven, quantitative metrics of FDG‐PET patterns outperformed MRI visual reads in predicting survival in this cohort. This may be due to the capacity of FDG‐PET to capture complex, dynamic patterns network failure optimally associated with clinical manifestations, whereas MRI reflects relatively static, already accumulated damage.

The diagnostic applications of FDG‐PET are well established in patients with dementia.^42^ The potential that FDG‐PET findings may also inform prognosis would further extend applications and indications for PET neuroimaging in patients with suspected prion disease. This could be particularly useful in atypical patients with disease courses that are either shorter or longer than predicted by current prognostication models combining clinical, imaging and genotype parameters.^24^, ^29^ If validated, integration of FDG‐PET findings in research settings could enhance the reliability of prognostic measures to support enrollment, stratification, and follow‐up in clinical trials of putative prion disease‐modifying therapies.

Although our relatively small sample size precluded consideration of the three‐way relationship between phenotypic heterogeneity, network physiology, and disease duration, our findings suggest that phenotypes selectively targeting the deep nuclei (e.g., fatal insomnia and motor variants) may have shorter symptomatic duration relative to those with predominant neocortical/cognitive (e.g., amnestic and language) variants. Indeed, qualitative inspection of Figures 2 and 3 indeed shows that patients with prion disease from the study cohort with cognitive variants (i.e., amnestic, language, and dysexecutive) tended to experience longer disease duration relative to those with motor, extrapyramidal, and global variants. These findings are consistent with prior observations emphasizing extended survival in patients with cognitive and affective presentations of CJD who often have cortical‐predominant findings on imaging.^25^, ^29^ FDG‐PET thus lends itself as an objective method of indexing such phenotypes facilitating clinical application and biological insights early in the disease course.

These findings must be interpreted in light of limitations. Patients with prion disease were identified within clinical settings. Thus, clinical and imaging assessments were not standardized across patients, and *PRNP* genotyping was not routinely performed. Molecular subtype is a strong predictor of disease duration in neuropathologic series,^26^, ^59^ and accounts for a substantial proportion of the variance in disease duration and patterns of neurodegeneration. In addition, the cohort was small and data‐driven clusters were even smaller. This reality precluded comprehensive evaluation of the relationships between molecular subtype, predominant phenotype, patterns of network degeneration, and survival time. This emphasizes the need for large‐scale studies with prospective recruitment and systematic collection of genetic data from participants. We did not find significant relationships between CSF markers and disease survival; this is inconsistent with recent, large‐scale studies assessing such associations.^4^, ^29^, ^49^ This unexpected finding suggests that we were underpowered to detect this established relationship. With this in mind, our ability to capture such relationships with FDG‐PET suggest that patterns of network degeneration may be a more robust marker of clinical outcomes and survival. Median time from symptom onset to FDG‐PET was relatively late. Although this is a reflection of current clinical care in patients with prion disease, prognostic models ideally would be validated closer to symptom onset. Overrepresentation of neocortical presentations may reflect recruitment bias, attributed to reliance on clinical FDG‐PET imaging, which is often obtained to inform the differential diagnosis of dementing disorders. Finally, prion disease continues to be disproportionately diagnosed in White individuals^25^; this cohort is no exception. Caution is advised before generalizing findings to patients from racial and ethnic backgrounds not represented in this cohort. These points emphasize the need to replicate these findings in larger cohorts, including diverse patients with representative prion disease presentations.

Our findings suggest that FDG‐PET may inform the relationship between affected neuroanatomy and survival in patients with prion disease. Patients with disproportionate hypometabolism in deep nuclei experienced a more aggressive disease course, whereas those with a predominant neocortical presentation had a more protracted course. If validated, these findings may identify the common pathways (i.e., neural networks) that predict survival in patients with prion disease, with the potential to inform clinical care and counseling at individual patient level and monitor progress/response in clinical trials of putative prion disease‐modifying therapies.

## Acknowledgements

We wish to express our gratitude to the patients and families for their participation. We also wish to thank all clinical and research staff involved in the care of the patients included in this study.

## Funding Information

Data collection was supported in part by a career development award from the National Institutes of Health/National Institute on Aging (K23 AG064029, GS Day). The Mayo Clinic Study of Aging is supported by the National Institutes of Health (U01 AG06786). This work was also supported by the Mayo Alzheimer's Disease Research Center (P30AG62677‐5), Consortium for Clarity in Alzheimer's Disease and Related Disorders Research Through Imaging (CLARiTI, DT Jones), and the Edson Foundation (DT Jones). The National Prion Disease Pathology Surveillance Center is funded by the Centers for Disease Control and Prevent (NU38CK000486).

## Conflict of Interest

Authors have no relevant conflict of interest in relation to this research.

## Author Contributions

NCL, DTJ, GSD contributed to the conceptualization and design of the study. NCL, YDP, BSA, DS, LRB, VG contributed to the acquisition and analysis of data. NCL, YDP, BSA, DS, LRB, VG, DTJ, GSA contributed to drafting the text or preparing the figures.

## Supporting information


Data S1.

## Data Availability

Anonymized study data will be shared pending review by the corresponding author of reasonable requests from qualified individuals.
