# Supplementary material for: FDG‐PET patterns associate with survival in patients with prion disease
Source: Ann Clin Transl Neurol. 2024 Oct 29;11(12):3227–37. doi: 10.1002/acn3.52230 (PMC11651209; doi:10.1002/acn3.52230)
Supplement: Supplementary file 1 — Data S1. [file ACN3-11-3227-s001.docx]

**Supplementary Materials**

| Supplementary Table 1. Characterization per molecular subtype | | | | | | | | |
| --- | --- | --- | --- | --- | --- | --- | --- | --- |
| Patient | Age at symptom onset | Age at FDG-PET | Sex | Months from FDG-PET to death | Clinical phenotype | FDG subtype | Molecular subtype | Histo-molecular subtype |
| 1 | 61.6 | 61.9 | Male | 4.2 | dysexecutive | Neocortical | E200K-129M | - |
| 2 | 76.9 | 77.4 | Female | 17.8 | dysexecutive | Transitional | MM2 | - |
| 3 | 66.5 | 66.6 | Female | 2.2 | global | Deep nuclei | MM1 | - |
| 4 | 66.5 | 67.6 | Female | 29.6 | visual | Neocortical | MM2 | - |
| 5 | 68 | 68.0 | Female | 1.0 | visual | Temporo-parietal | MM1 | - |
| 6 | 33.4 | 35.2 | Female | 22.3 | psychiatric | Transitional | MM2 | - |
| 7 | 68.6 | 69.1 | Male | 7.0 | cerebellar | Deep nuclei | VV2 | - |
| 8 | 64.7 | 66.4 | Male | 33.0 | visual | Temporo-parietal | MM1-2 | - |
| 9 | 53.6 | 53.8 | Male | 2.9 | global | Temporo-parietal | E200K-129M | - |
| 10 | 75.8 | 76.0 | Male | 2.3 | global | Transitional | MM1 | - |
| 11 | 62.1 | 62.4 | Female | 5.4 | cerebellar | Deep nuclei | VV1-2 | - |
| 12 | 35 | 36.5 | Male | 24.6 | hyperkinetic | Transitional | MM2 | - |
| 13 | 60.8 | 61.0 | Female | 3.4 | corticobasal | Transitional | MM1 | - |
| 14 | 56.3 | 58.6 | Female | 28.9 | dysexecutive | Temporo-parietal | MV1-2 | MV1-2C |
| 15 | 65.4 | 65.6 | Female | 5.9 | cerebellar | Transitional | VV2 |  |
| 16 | 51.6 | 52.2 | Female | 26.8 | dysexecutive | Neocortical | MV2 | MV2K |
| 17 | 61.7 | 63.6 | Male | 35.0 | amnestic | Temporo-parietal | MV2 | MV2C |
| 18 | 58.7 | 59.6 | Male | 14.1 | global | Temporo-parietal | MV2 | MV2C |
| FDG-PET = Fluorodeoxyglucose positron emission tomography. | | | | | | | | |


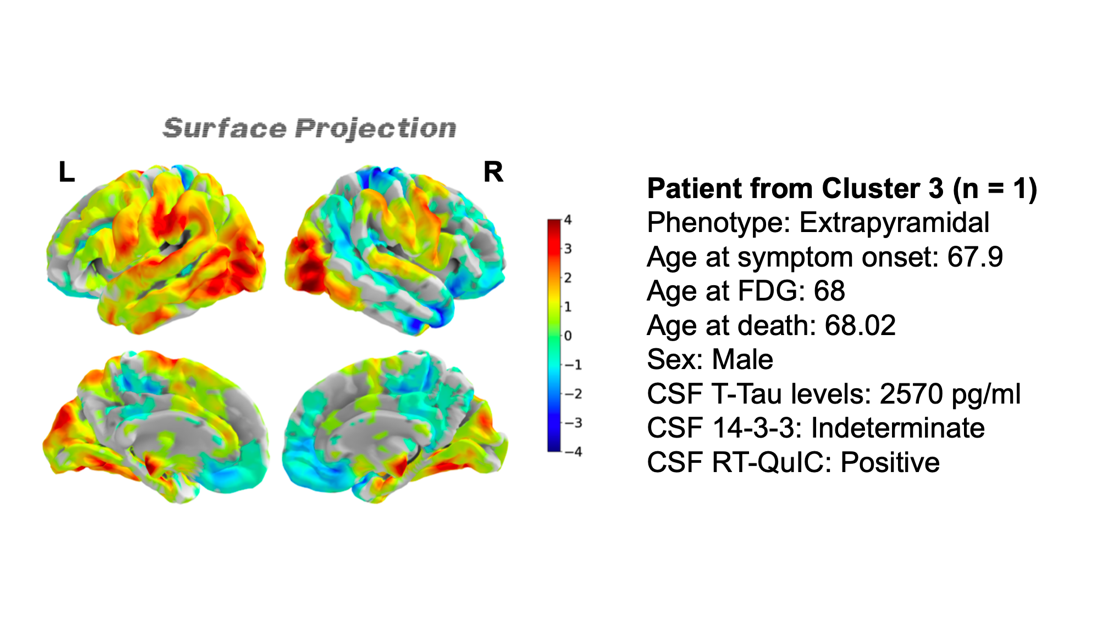


**Supplementary Figure 1.** Individual data from the patient included in Cluster 3. This patient had a predominant extrapyramidal phenotype and exhibited a mixed pattern of hypermetabolism in posterior occipital and parietal area (left more than right) and hypometabolism in the right temporal and bilateral orbitofrontal areas. The pattern of whole-brain metabolism and resulting *Z* scores were generated using an internal clinical tool including a database of thousands of FDG-PET images. FDG = Fluorodeoxyglucose; T-Tau = Total-Tau; pg/ml = picogram per millimeter.


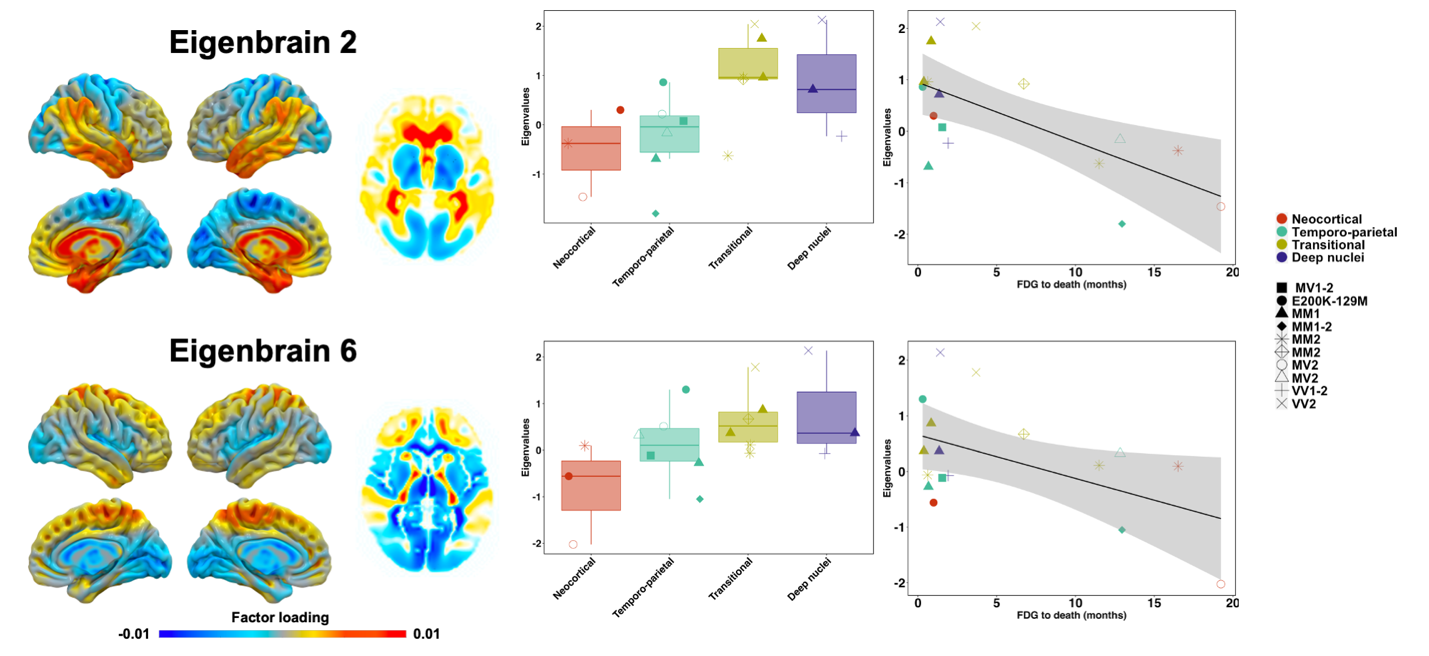


**Supplementary Figure 2.** Comparisons of data-driven metabolic clusters on eigenbrains according to molecular subtype. The eigenbrains were derived using an independent dataset of research patients as described in Jones et al. (2022). The color bars represent positive (warm colors) and negative (cold colors) loadings associated with each eigenbrain. The eigenbrains reflect relative metabolism between two sets of brain areas, and the directionality (positive and negative) is arbitrary. For both eigenbrains, higher eigenvalues are indicative of lower metabolism in cold colors relative to warm colors, and vice-versa. Only eigenbrains showing significant differences across clusters are shown. Dot shapes indicate molecular subtype.
